# Renal ultrasonography in healthy Saanen goats: topographic, biometric and echotextural assessment

**DOI:** 10.1007/s11259-026-11109-3

**Published:** 2026-03-03

**Authors:** Tatiane Vitor da Silva, Isabela Bernardes Moreira, Raissa da Silva Carvalho, Clara Beatriz de Avila Santiago, Mário Felipe Alvarez Balaro

**Affiliations:** 1 Faculdade de Veterinária, Universidade Federal Fluminense, Av. Vital Brasil Filho, 64, Niterói, CEP 24230-340 RJ Brazil; 2 Universidade Federal de Minas Gerais, Av. Presidente. Antônio Carlos, Pampulha, 6627, Belo Horizonte, CEP 31270-901 MG Brazil

**Keywords:** Abdominal ultrasonography, B-mode ultrasound, Renal biometry, Small ruminant, Ultrasound imaging

## Abstract

This study aimed to establish reference ultrasound values for renal dimensions in healthy dairy goats and to investigate correlations between kidney size and body measurements. Thirty-four clinically healthy lactating Saanen goats underwent standardized ultrasonographic evaluations. The left kidney showed significantly greater length, height, and medullary pyramid dimensions than the right (*p* < 0.05). No significant differences were observed for other variables (*p* > 0.05). The right kidney was located in the craniodorsal quadrant between the 12th and 11th intercostal spaces, while the left kidney was primarily positioned in the right caudodorsal quadrant. Mean kidney volume was 68.91 ± 9.71 cm³ for the right kidney and 63.32 ± 22.31 cm³ for the left. Cortical and medullary diameters averaged 0.80 ± 0.13 cm and 0.81 ± 0.15 cm, respectively. Ureter, renal pelvis, and hilum measurements were 0.11 ± 0.03 cm, 0.20 ± 0.04 cm, and 0.60 ± 0.12 cm, respectively, for both kidneys. Weak correlations were found between body biometric parameters and renal dimensions. These findings underscore the importance of species-specific ultrasonographic reference values in small ruminants to enhance diagnostic precision and support the early detection of renal disorders.

.

## Introduction

The kidneys play a fundamental role in maintaining homeostasis by regulating water balance, electrolyte levels, and the excretion of metabolic waste products from the body (Cesta 2006; Trefts et al. 2017). However, these organs are susceptible to a range of pathological conditions that can alter their size and structure.

Detailed clinical examination of any presented for disease diagnosis and treatment is essential. However, on physical examination assessment of the kidney size and alterations in its parenchymal architecture is not possible. B-mode ultrasonography is a valuable diagnostic modality that can provide real‑time information on renal size, shape, position, surrounding structures, and internal echotexture (Steininger and Braun 2012; Stankiewicz et al. 2023; Tiryaki and Aksu 2024).

Although renal ultrasound is widely used in veterinary medicine, its application in small ruminants remains underutilized. In contrast to companion animals and humans where renal ultrasonographic landmarks are well established data for goats are scarce and often lack consistency across breeds and studies. The limited literature regarding anatomical and Doppler studies in goats, highlight variability and underscore the need for standardized protocols (Marzok and Tharwat 2025).

To address this gap, providing reliable ultrasonographic parameters is essential to improve the accuracy of renal assessments in both clinical and research contexts. Therefore, the objective of this study was to establish reference values for renal dimensions in healthy dairy goats and also to explore the possibility of any correlations between kidney size and the body measurements.

## Materials and methods

### Study location and experimental conditions

The study was conducted on a commercial dairy goat farm located in Sapucaia, Rio de Janeiro, Brazil. The entire flock was vaccinated against Clostridiosis and Rabies and dewormed according to the local herd health plan. The goats managed under an intensive production system were fed four times daily a Total Mixed Ration (TMR). Fresh drinking water and mineral salt formulated specifically for goats were provided *ad libitum*.

### Animals and health status

A total of 34 lactating Saanen goats were included in the study (3.75 ± 1.0 years of age; 2.6 ± 0.8 body condition score; 53.3 ± 9.4 kg live weight; 254 ± 89.5 days in lactation; and 2.5 ± 0.8 L of daily milk production). To ensure animal health, all goats underwent a clinical examination as described by Pugh et al. (2020), in addition to laboratory testing of their blood samples for complete blood count and renal and hepatic biochemistry. The complete blood count was performed using an automated hematology analyzer (BC-2800Vet; Mindray, São Paulo, SP, Brazil). Biochemical analysis was conducted using a spectrophotometer and commercial reagent kits., The enzymatic-colorimetric assessment of urea; kinetic-colorimetry for creatinine, aspartate aminotransferase (AST), alkaline phosphatase (AP), total protein (TP), and albumin were all carried out. Globulin concentration was calculated by subtracting albumin from total protein (TP – albumin) (Thrall et al. 2012).

### Body biometric measurements

Body biometric measurements including thoracic perimeter, abdominal perimeter, height at the withers, and body length were - analyzed for possibility of any correlations with linear renal dimensions (kidney length, height, width, hilum thickness, pelvis thickness, and ureter diameter and renal volume).

### B-mode ultrasonography

**Fig. 1:**
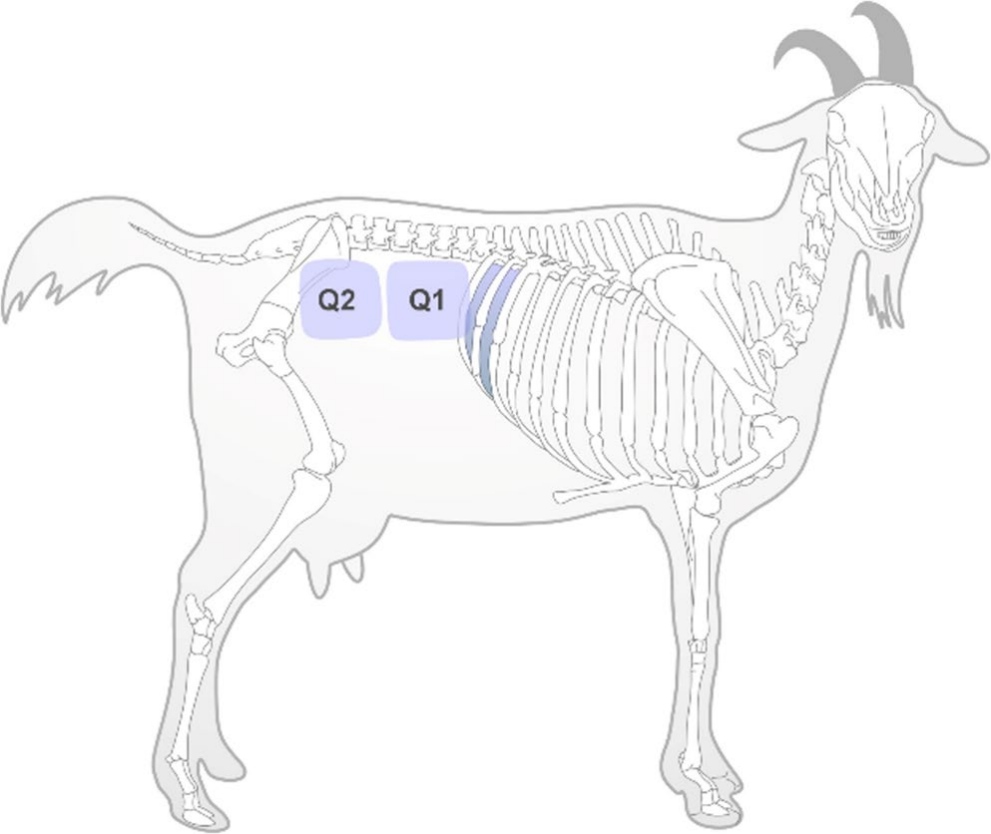
Schematic representation of the evaluated sites in the renal ultrasound examination of goats. The gray band marks the 12th and 11th intercostal spaces. Imaginary quadrants Q1 and Q2 are indicated. (Adapted from Steininger and Braun 2012). Drawing credit: Guilbert Araujo

All ultrasound examinations were performed by the same technician, every animal evaluated only once. A portable B-mode ultrasound device (Sonoscape S6; SonoScape, Shenzhen, China) equipped with a 5.0 MHz convex transducer was used. Equipment settings were standardized and remained constant throughout the study (frames per second: 28; depth: 8.9 cm; gain: 146; power: 80%; focus: 2–3 cm). The scanning procedures were carried out over ten consecutive days to evaluate all the 34 goats. During USG examination, animals were held standing without sedation,

For kidney evaluation and localization, the right and left flanks were divided into two imaginary quadrants: craniodorsal (Q1) and caudodorsal (Q2). Additionally, the region corresponding to the 11th and 12th intercostal spaces was also examined, as per the techniques described by Steininger and Braun (2012) (Fig. 1).

Both the kidneys were imaged in longitudinal and transverse planes. In longitudinal views, kidney length and height were measured as the distances between the cranial and caudal poles and between the dorsal and ventral surfaces, respectively. Renal cortical thickness (RCT) was determined by drawing a perpendicular line from the renal capsule to the base of the medullary pyramid. Corticomedullary thickness was measured from the renal capsule to the renal sinus. Transverse images were used to measure kidney width and the diameters of the renal pelvis, ureter, and hilum.

Medullary thickness was calculated by subtracting cortical thickness from corticomedullary thickness. The corticomedullary ratio was obtained by dividing cortical thickness by medullary thickness. The diameters of the three largest visible medullary pyramids were recorded by placing the caliper along their longest axes. Renal volume was estimated using the ellipsoid formula: Volume = length × width × thickness × π / 6. A comparative analysis of renal and hepatic echogenicity was also performed.

### Statistical analysis

Pearson’s correlation coefficient was used to assess associations between body biometric variables and renal biometric measurements. The effect of laterality (left vs. right kidney) on renal measurements was analyzed using an unpaired t-test. Statistical significance was set at *P* < 0.05, and trends were noted for values between 0.05 and 0.10.

### Limitations of the study

The number of animals used, is a limitation of this study. this is justified because the breeds are expensive and also because of the long and time-consuming bureaucratic process for acquiring more animals. For this reason, we emphasize that our results serve as a basis for other similar studies with a larger sample of animals and different breeds.

## Results

### Laboratorial exams

Hematological and biochemical parameters of the goats were within the species’ reference ranges. The values obtained were: urea 42.1 ± 10.8 mg/dL; creatinine 0.6 ± 0.1 mg/dL; aspartate aminotransferase (AST) 102.4 ± 20.1 IU/L; alkaline phosphatase (ALP) 249.3 ± 64.0 IU/L; total protein 7.1 ± 0.7 g/dL; albumin 3.0 ± 0.2 g/dL; globulin 4.02 ± 1.0 g/dL; calcium 8.63 ± 1.3 mg/dL; and phosphorus 7.82 ± 2.57 mg/dL (Kaneko et al. 2008).

### Ultrasound findings and biometric correlations

The right kidney was located in the craniodorsal quadrant (Q1) in 82.4% (28/34) of the animals and within the 12th and 11th intercostal spaces in 91.2% (31/34) and 11.8% (4/34), respectively, adjacent to the caudate lobe of the liver. The left kidney was observed in the right caudodorsal quadrant (Q2) in 97.1% (33/34) of the goats, while in 2.9% (1/34), it was located in the left caudodorsal quadrant.

**Fig. 2:**
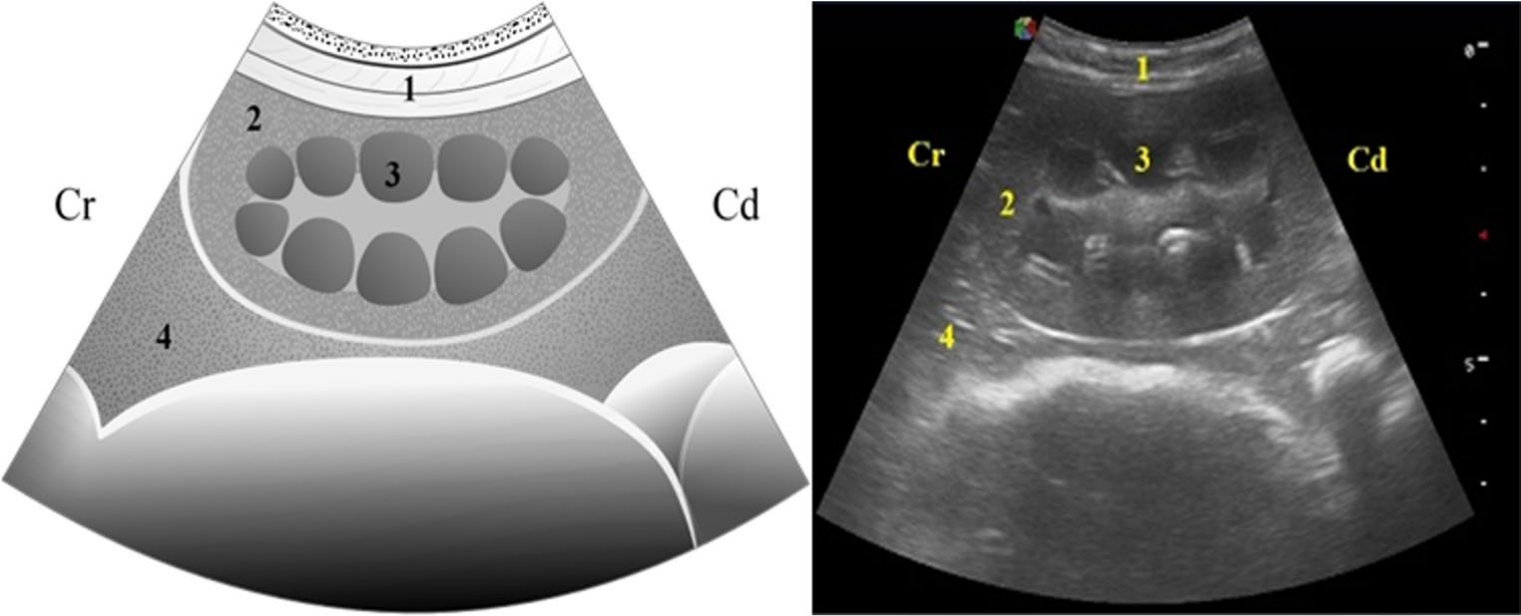
Graphic representation (left) and real image (right) of the ultrasonographic appearance of the kidney in a longitudinal Sect. (1) Lateral abdominal wall; (2) Renal cortex; (3) Medullary pyramid (4) Liver parenchyma; Cr: Cranial; Cd: Caudal (5.0 MHz convex transducer; depth: 7 cm). Drawing credit: Guilbert Araujo

In longitudinal sections, the kidneys appeared elongated and surrounded by a hyperechoic capsule. The cortex was homogeneous and hypoechoic, while the medullary region displayed multiple round to oval, anechoic medullary pyramids (Fig. 2).

In comparison to the liver, the kidneys were isoechoic in 29.41% (10/34) and hypoechoic in 70.59% (24/34) of the animals.

**Fig. 3:**
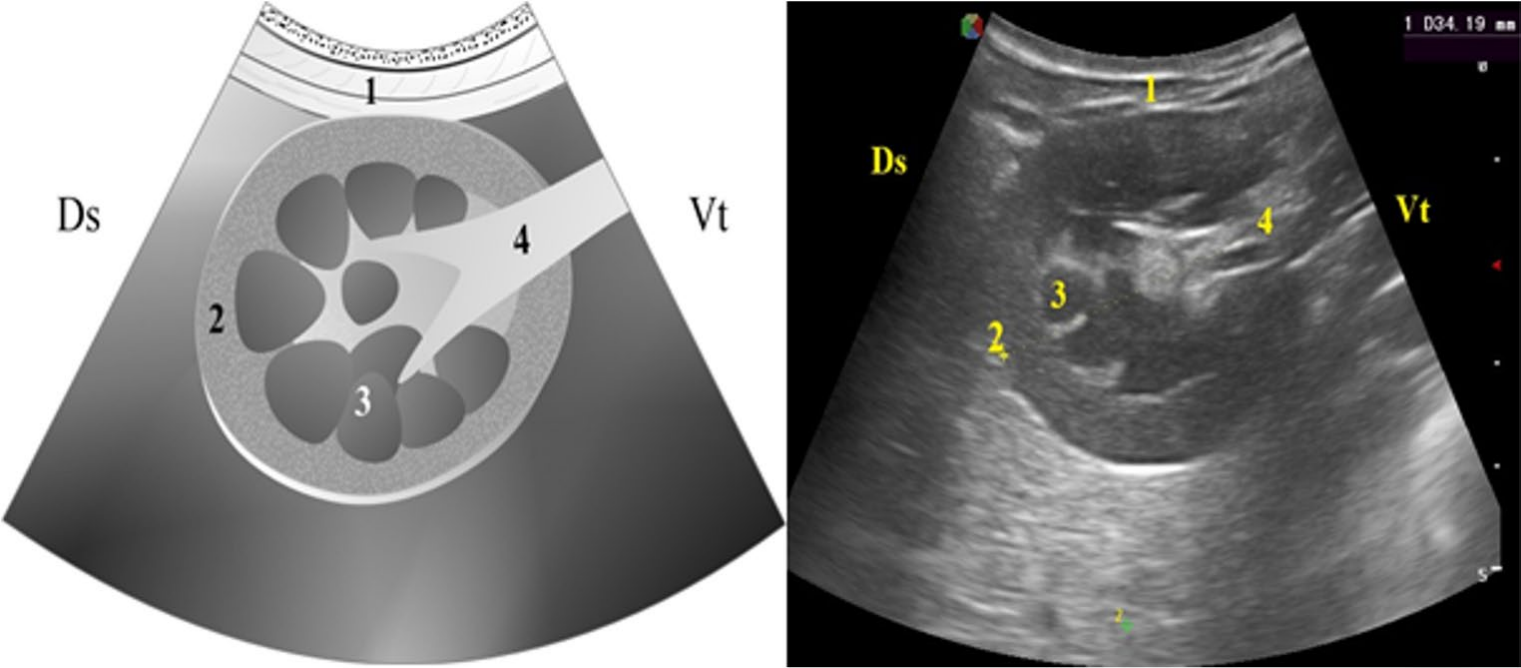
Graphic representation (left) and real image (right) of the ultrasonographic appearance of the kidney in a cross-Sect. (1) Lateral abdominal wall; (2) Renal cortex; (3) Renal medulla; (4) Renal hilum (5.0 MHz convex transducer; depth: 5 cm). Drawing credit: Guilbert Araujo

In cross-sectional views, the kidneys exhibited a rounded shape. The renal pelvis, which assumed a “Y” configuration, was visible, and the hilum appeared as a hyperechoic band from which the renal artery, vein, and ureter emerged. The ureter was seen as a thin hyperechoic line without a distinguishable lumen (Fig. 3).

In all animals, the renal cortex and medulla were clearly distinguishable, indicating a preserved corticomedullary relationship. The left kidney showed significantly greater values for length, height, and medullary pyramid dimensions compared to the right kidney (*p* < 0.05). Other variables including width, volume, ureter diameter, renal pelvis thickness, and medullary pyramid size did not differ significantly between sides (*p* > 0.05) (Table 1).

**Table 1:** Renal biometry, mean ± standard deviation and (minimum and maximum) values in 34 healthy Saanen goats

Variable	Right kidney	Left kidney	*P* value
Length (cm)	6.52 ± 0.53 (5.51–7.46)	7.35 ± 0.46 (6.02–8.13)	< 0.05
height (cm)	4.12 ± 0.41 (3.25–5.03)	3.92 ± 0.55 (2.91–4.92)	< 0.05
Thickness (cm)	4.82 ± 0.42 (4.02–5.87)	4.74 ± 0.61 (3.41–6.01)	> 0.05
Volume (cm^3^)	68.91 ± 9.71 (50.21–86.12)	63.32 ± 22.31 (48.20–85.11)	> 0.05
Cortical thickness (cm)	0.80 ± 0.13 (0.52–1.10)	0.81 ± 0.15 (0.31–1.10)	> 0.05
Medullary thickness (cm)	0.81 ± 0.21 (0.53–1.61)	0.83 ± 0.15 (0.55–1.02)	> 0.05
Corticomedullary (cm)	1.61 ± 0.22 (1.13–2.14)	1.63 ± 0.23 (1.21–2.12)	> 0.05
Corticomedullary ratio (cm)	1.0 0 ± 0.20 (0.52 − 1.21)	1.0 0 ± 0.27 (0.31–1.21)	> 0.05
Medullary pyramid 1 (cm)	0.88 ± 0.15 (0.53–1.2)	0.91 ± 0.17 (0.61–1.6)	< 0.05
Medullary pyramid 2 (cm)	0.80 ± 0.15 (0.56–1.13)	0.86 ± 0.16 (0.54–1.11)	< 0.05
Medullary pyramid 3 (cm)	0.70 ± 0.14 (0.44–1.01)	0.77 ± 0.16 (0.51–1.11)	< 0.05
Ureter diameter (cm)	0.11 ± 0.03 (0.06–0.18)	0.11 ± 0.03 (0.06–0.21)	> 0.05
Pelvis diameter (cm)	0.20 ± 0.04 (0.1–0.29)	0.20 ± 0.04 (0.13–0.2)	> 0.05
Hilum diameter (cm)	0.61 ± 0.12 (0.41–0.81)	0.63 ± 0.12 (0.42–0.83)	> 0.05

Weak positive correlations were found between body biometric parameters and renal measurements. Thoracic perimeter showed weak correlations with hilum thickness (*r* = 0.296) and medullary pyramid size (*r* = 0.247). Abdominal perimeter correlated weakly with kidney width (*r* = 0.227), renal volume (*r* = 0.244), ureter diameter (*r* = 0.255), and medullary pyramid size (*r* = 0.273). No significant correlations were observed between kidney measurements and either body length or height at the withers (*p* > 0.05).

## Discussion

This study establishes preliminary biometric reference values for dairy goats of the Saanen breed. Both kidneys were successfully visualized in all goats, with predominance on the right side. Although both kidneys are typically located on the right due to presence of the rumen, one animal had its left kidney in the left side. This variation is not indicative of pathology, as the left kidney in some animals stay dorsal to the rumen rather than getting shifted to the right side (Ferreira et al. 2014; Steininger and Braun 2012).

The ultrasound images obtained in longitudinal and transverse planes were consistent with findings in previous studies involving goats and sheep. The similarities observed among these species can be attributed to their comparable anatomical and physiological characteristics, particularly in relation to the size, shape, and internal organization of the renal parenchyma (Steininger and Braun 2012; Santarosa et al. 2016).

In the present study, the left kidney was significantly longer than the right, contrasting with the findings of Steininger and Braun (2012), who reported no difference in the length between kidneys of the two sides in Saanen goats. Asymmetry in renal dimensions has also been reported in neonatal lambs (Stankiewicz et al. 2023). Anatomical positioning may account for this difference: the right kidney lies adjacent to the caudate lobe of the liver, limiting its mobility and expansion, whereas the left kidney, located in a more spacious vacant region of the abdominal cavity, may grow larger or appear larger on ultrasound due to fewer imaging artifacts (Lima et al. 2008). From a clinical perspective, recognizing this asymmetry is important to avoid misinterpretation of physiological variations as pathological changes, such as unilateral renal enlargement or atrophy. Thus, knowledge of these species-specific differences contributes to a more accurate assessment of renal morphology in small ruminants.

Renal length is a key ultrasonographic parameter for evaluating urolithiasis because obstruction of the urinary tract can impair urine outflow, leading to increased intrapelvic pressure, hydronephrosis, and subsequent renal enlargement (Braun et al. 1992; Ferreira et al. 2014). However, the diagnostic value of this parameter depends on the stage and severity of the condition. In advanced cases, the affected kidney may become enlarged due to sustained pressure changes, whereas in experimentally induced urolithiasis, renal size often remains within normal limits, as reported by Santarosa et al. (2016). Therefore, slight differences in renal dimensions should be interpreted with caution, since mild asymmetry may reflect normal anatomical variation, whereas marked or progressive enlargement can be clinically relevant, indicating obstruction, compensatory hypertrophy, or other underlying pathology.

The echotexture of the renal cortex and medulla observed in this study aligns with that reported in healthy humans and animals, demonstrating a homogeneous cortical pattern and clear corticomedullary distinction across species. This similarity is clinically relevant because alterations in cortical echogenicity or loss of corticomedullary definition are frequently associated with renal disease processes, including chronic nephropathy, interstitial nephritis, and glomerulonephritis. Therefore, assessment of cortical and medullary echotexture provides an important parameter for distinguishing normal renal architecture from pathological changes in both veterinary and human medicine (Braun et al. 2013; Stankiewicz et al. 2023; Tiryaki and Aksu 2024).

This difference reflects the higher cellular density of the cortex versus the tubular content of the medulla. Evaluation of cortical echogenicity is clinically relevant, as increased echogenicity or cortical thinning may suggest renal pathology (Scott 2013).

Variability in renal echogenicity relative to the liver was observed among animals. This parameter is clinically relevant, as increased renal echogenicity can be associated with chronic kidney disease, glomerulopathies, infections, or fibrosis. In all cases, the corticomedullary distinction was preserved, which represents an important indicator of normal renal architecture. Disruption of this ratio is often linked to chronic kidney disease (CKD). Collectively, these findings reinforce the value of incorporating renal morphological assessment into routine ultrasonographic screening (Floeck 2009; Moela et al. 2016; Perondi et al. 2020; Tharwat 2021).

Measurements of the renal hilum, pelvis, and ureter obtained in transverse images are not commonly reported in the literature for small ruminants. Some authors suggest that the ureter, renal vein, and artery can only be differentiated via Doppler imaging (Steininger and Braun 2012). However, in this study, the ureter was clearly visualized as a hyperechoic linear structure continuous with the renal pelvis. These findings provide reference values that may aid in identifying the clinical cases of hydronephrosis, where in dilation of the renal pelvis and ureter is commonly noticed (Scott 2013).

Medullary pyramids exhibited variation in size across animals included in this trial. It may reflect the normal anatomical individual difference. However, enlargement or compression of pyramids may also result from urinary tract obstruction, emphasizing the need for knowing their measurements in healthy animals that could help in early diagnosis of the disease (Floeck 2009; Tharwat 2021).

The weak positive correlations observed between body biometric parameters and renal measurements suggest that overall body size has a limited influence on renal dimensions. No significant associations were found between renal measurements and body length or height at the withers, indicating that these specific biometric parameters do not significantly affect renal morphology. Clinically, this suggests that renal ultrasound assessments can be interpreted largely independently of overall body size.

One limitation of this study is the exclusive inclusion of lactating Saanen goats from a single dairy flock, which may restrict the generalizability of the findings to other breeds, ages, or physiological stages. Additionally, inter-operator variability was not assessed, although all exams were performed by the same experienced technician to ensure consistency. Therefore, future studies should explore the applicability of these reference values across different goat breeds and physiological conditions (e.g., pregnancy, early lactation, or aging). Incorporating Doppler ultrasonography or histopathological correlations would enhance the clinical relevance and diagnostic specificity of ultrasonographic findings in caprine nephrology.

Transabdominal ultrasonography in standing, unseated goats was feasible and satisfactory for evaluating renal morphology and dimensions. These echodescription and measurement values may serve as base for comparison in clínical cases and may also support future research on nephropathies, that could subsequently enhanc veterinary care and flock health management.

## Data Availability

The datasets generated during the current study are available from the corresponding author on request.
